# Subretinal leakage of a retinal capillary macroaneurysm - a case report

**DOI:** 10.1186/s12886-021-01984-6

**Published:** 2021-05-17

**Authors:** Ahmad S. Rehmani, Touka Banaee, Fuad Makkouk

**Affiliations:** University of Texas Medical Branch, 700 University Boulevard, Galveston, Tx 77555 USA

**Keywords:** Capillary macroaneurysm, External limiting membrane, Case report

## Abstract

**Background:**

Report a rare case of retinal capillary macroaneurysm with associated subretinal fluid.

**Case presentation:**

A 71-year-old male underwent full ophthalmic examination including Optical Coherence Tomography (OCT), Fluorescein Angiography (FA). Fundus examination showed moderate non-proliferative diabetic retinopathy of both eyes with scattered microaneurysms. On initial visit, FA displayed a hyperfluorescent lesion with leakage on late frames in the left eye. OCT revealed the lesion to be spheroid with a hyperreflective wall and hyporeflective lumen in the inner retina, corresponding to a capillary macroaneurysm. Intraretinal cystic fluid surrounded the lesion. On subsequent visit 7 months later, subretinal fluid in the location of the capillary macroaneurysm was noted on OCT. Vision was maintained at 20/30–2 OD, 20/40 OS throughout. No treatment was necessary.

**Conclusion:**

Subretinal fluid from the capillary macroaneurysm likely developed from its juxtafoveal location and discontinuity of the external limiting membrane (ELM); a barrier preventing flow of intraretinal fluid to the outer retina.

## Background

Aneurysms are defined as fusiform or round dilatation, otherwise known as outpouching, of vasculature. Aneurysms of retinal vessels can involve all aspects of the circulation and may develop from arteries, veins, collateral circulation or capillaries [1]. When the complexes are large and involve the first three orders of the arterial circulation, they have been termed retinal arterial macroaneurysms (RAM) [1, 2]. These aneurysms boast a thickened wall and are often associated with hypertension [2]. Smaller aneurysms, known as microaneurysms, usually involve capillaries and are associated with ischemic conditions such as diabetes and retinal vein occlusions [3]. Large dilations of the capillary system, known as retinal capillary macroaneurysms, have also been described; defined in size as larger than 100–300 μm depending on the source [3–7]. While RAMs can cause subretinal, intraretinal and preretinal hemorrhage, exudation and fluid, subretinal fluid in capillary macroaneurysms is rare. Herein we report a unique case of a juxta-foveal capillary macroaneurysm associated with subretinal fluid.

## Case presentation

A 71-year-old male, followed for 5 years for diabetes mellitus presented to the University Eye Clinic for yearly examination. Patient had no complaints including blurry vision, metamorphopsias or scotomas. He had a history of stable, well-controlled diabetes mellitus 2 with a hemoglobin A1c of 6.5 the month before. On record, patient never had a hemoglobin A1c above 7.0. He also had a past medical history of hypertension, hyperlipidemia and prostate cancer 19 years prior which was cured after a complete resection. His medications included atorvastatin, metformin, glimepiride and losartan. On examination his best corrected visual acuity was 20/30–2, 20/40–2 and intraocular pressure 11, 14 mmHg in the right and left eyes respectively. Pupillary examination, extraocular muscles and confrontation visual fields were all within normal limits. Slit lamp examination showed a right upper eyelid nevus and bilateral nasal pterygium stable for many years.

**Fig. 1:**
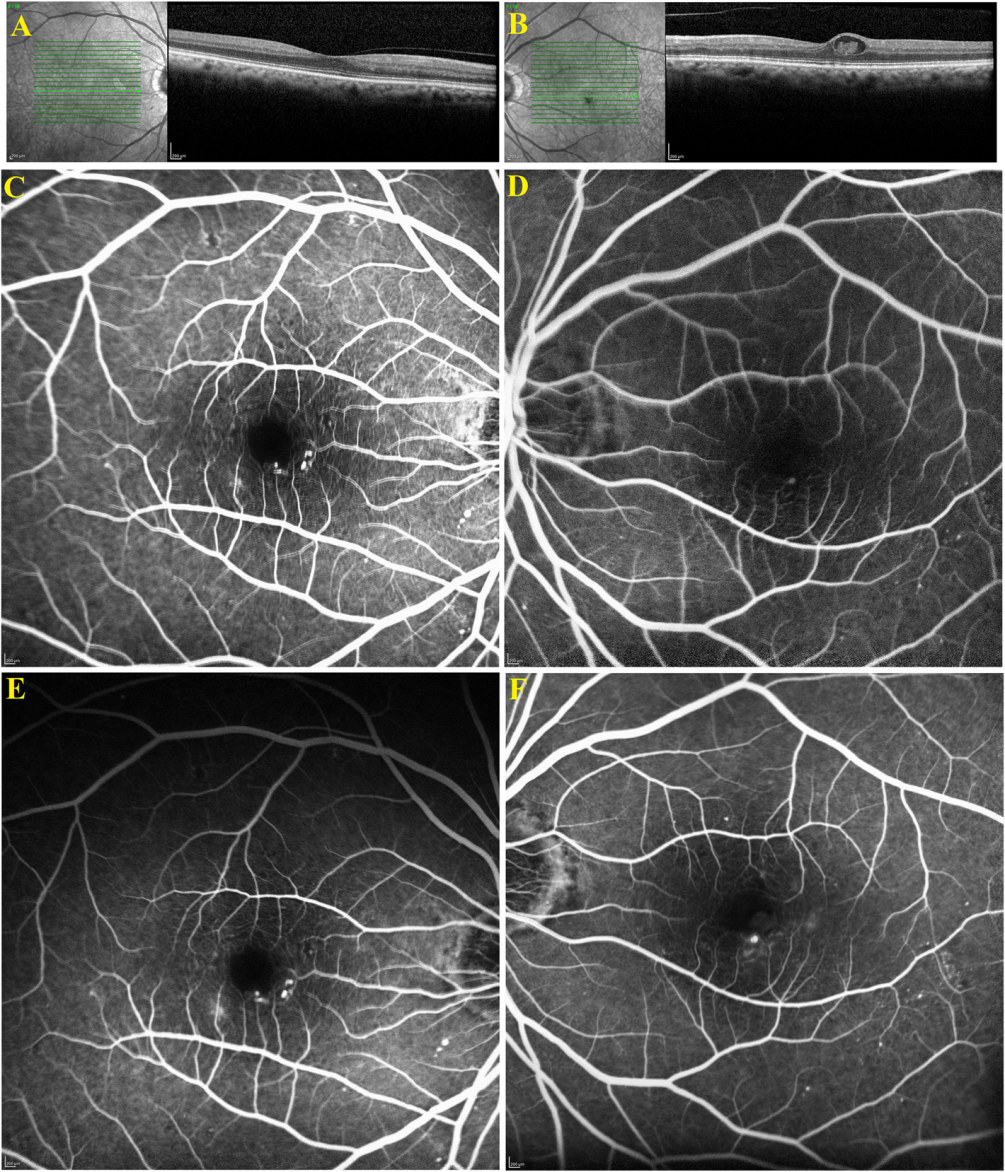
Optical Coherence Tomography (OCT) fluorescein angiography (FA) of the right eye (**a**, **c**, **e**) and left eye (**b**, **d**, **f**) at initial visit. OCT of the right eye (**a**) is normal. OCT of the left eye (**b**) revealed a spheroid lesion measuring 381 μm with a hyperreflective wall and hyporeflective lumen harboring some irregular hyperreflective content in the inner retina. The hyaloid is lifted. FA of the right eye at 33 s (**c**) and 3 min (**e**) show scattered microaneurysms seen as pinpoint hyperfluorescence inferonasal and inferior to the fovea. FA of the left eye at 30 s (**d**) reveals a hyperfluorescent sub foveal lesion inferior to the fovea with subsequent leakage and staining at 3 min (**f**) as well as scattered microaneurysms particularly along the temporal macula seen as pinpoint hyperfluorescence

On fundus examination of the left eye, a large juxta-foveal aneurysm with surrounding intraretinal fluid was present inferior to the fovea. Both eyes had scattered microaneurysms and mild peripheral retinal degeneration nasally. Initially, Optical Coherence Tomography (OCT) of the right eye was normal (Fig. 1a). The left eye revealed a spheroid lesion in the inner retina with a hyperreflective wall and hyporeflective lumen harboring some irregular hyperreflective content. The hyaloid was lifted. (Fig. 1b). Fluorescein angiography (FA) of both eyes revealed scattered microaneurysms in early frames (Fig. 1c, d). The left eye revealed a large, hyperfluorescent lesion inferior to the fovea with associated leakage in late frames (Fig. 1d, f). As patient’s vision was unaffected, he was recommended to return in 3 months’ time. Due to social distancing regulations from the coronavirus pandemic, patient had a tele-visit at that time where he reported no visual changes.

**Fig. 2:**
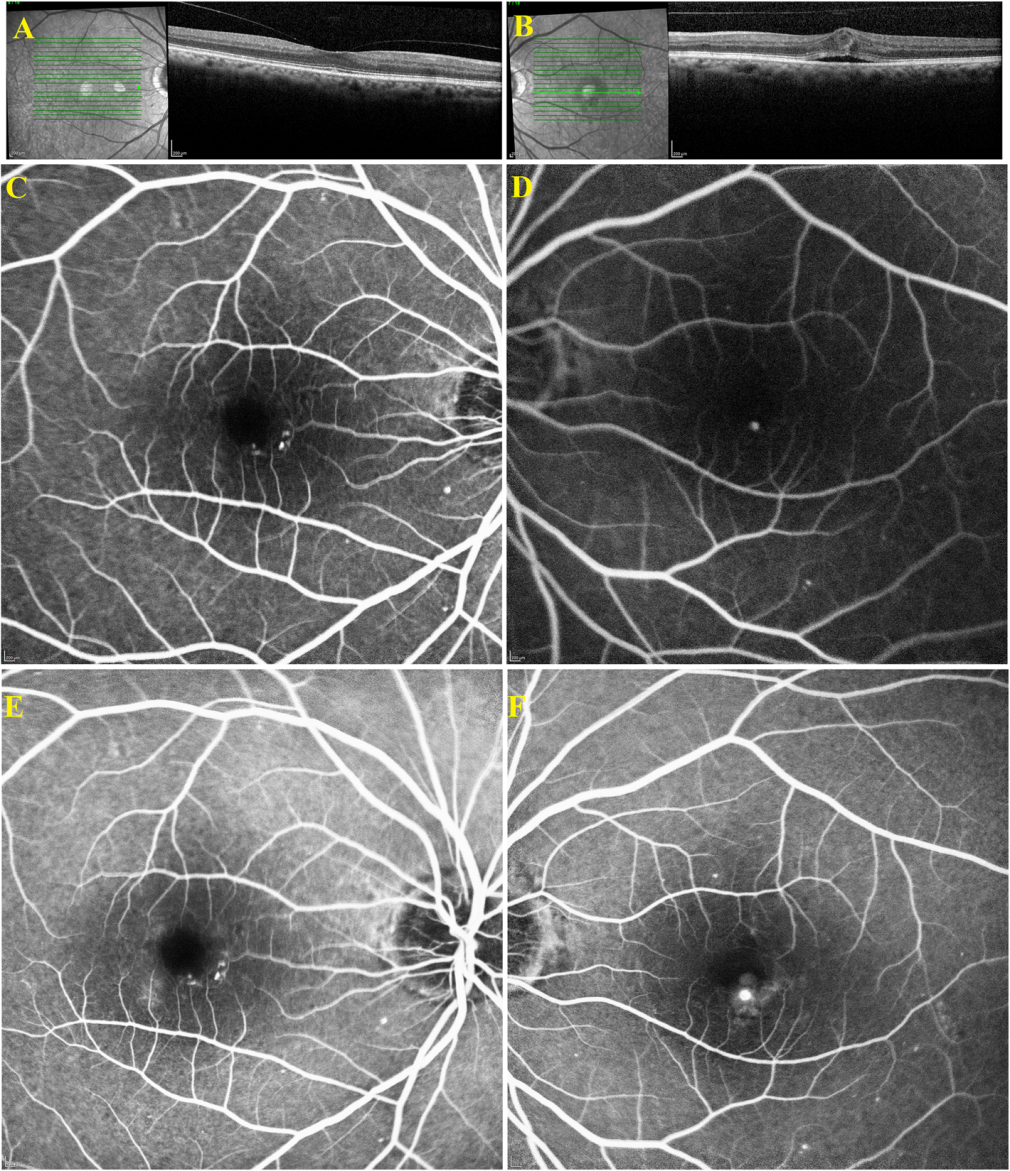
Optical Coherence Tomography (OCT) fluorescein angiography (FA) of the right eye (**a**, **c**, **e**) and left eye (**b**, **d**, **f**) at follow-up visit. OCT of the right eye (**a**) is normal. OCT of the left eye (**b**) shows a spheroid lesion with a hyperreflective wall, relatively dark lumen, intraretinal cystic spaces and subretinal fluid. The hyaloid is lifted. FA of the right eye at 30 s (**c**) and 3 min (**e**) show scattered microaneurysms seen as pinpoint hyperfluorescence inferonasal and inferior to the fovea. FA of the left eye at 33 s (**d**) reveals a hyperfluorescent lesion inferior to the fovea with subsequent leakage and staining at 3 min (**f**) as well as scattered microaneurysms particularly along the temporal macula seen as pinpoint hyperfluorescence

On subsequent in-person visit, 7 months after initial visit, patient reported no visual changes and maintained a visual acuity of 20/30–2 and 20/40–2 of the right and left eyes respectively. OCT of the right eye was normal (Fig. 2a). OCT of the left eye revealed a spheroid lesion with a hyperreflective wall, relatively dark lumen, intraretinal cystic spaces and new subretinal fluid. The hyaloid was lifted. (Fig. 2b). FA of both eyes revealed scattered microaneurysms. (Fig. 2c, d) The left eye revealed a large, hyperfluorescent lesion inferior to the fovea with leakage and staining that appeared significantly more hyperfluorescent in late frames from prior FA (Fig. 2e, f). Follow-up visits at 12- and 18- months revealed a stable lesion with resolving intraretinal and subretinal fluid and no change in visual acuity. Patient continued to be closely monitored without treatment.

## Discussion and conclusions

The etiology of capillary macroaneurysms is largely unknown. The formation of capillary microaneurysms involves selective pericyte loss, smooth muscle cell death and localized increases in capillary hydrostatic pressure. Some evidence shows that capillary macroaneurysms are microaneurysms that enlarge beyond a threshold size where they develop a thickened wall and characteristic lobular shape [6, 8]. In vivo adaptive optics imaging supports this claim by showing an increase in vessel wall thickness with increase in diameter of aneurysms. As such, larger aneurysms had partial fill with fluorescein on OCT angiography possibly due to parietal thickening from the vessel wall [9]. Matrix metallopeptidase 9 (MMP-9), which functions to break down basement membrane proteins, has been shown to be present in walls of larger microaneurysms; suggesting there is potentially even less resistance to expansion beyond a definite aneurysm size [10]. However, it is not known which microaneurysms will undergo a transformative expansion. Paques et al. defined macroaneurysms based on therapeutic outcomes. Lesions smaller than 130 μm reported limited visual improvement with focal laser treatment and were termed microaneurysms whereas larger, more laser-susceptible lesions, were termed macroaneurysms [6]. Yet other studies describe the development of macroaneurysms after branch vein occlusions, suggesting pathogenesis related to retinal ischemia and pressure increase in the capillary net [4, 7]. Farias, et al. went so far as to rename capillary macroaneurysms as telangiectatic capillaries (TC) to suggest they are complex, large microvascular abnormalities to prevent confusion with RAMs. Like others, however, the authors suggest TCs are a continuum rather than a strict dichotomy, when compared with capillary microaneurysms. Farias, et al. recommend imaging of the lesions as the best method to elucidate their characteristics [5]. Indocyanine Green (ICG) and Optical Coherence Tomography (OCT) are the best imaging modalities; although FA may be diagnostic when other exam findings favor the diagnosis [6, 11].

A unique characteristic of RAMs is the potential presence of edema/hemorrhage in multiple retinal layers i.e. subretinal, intraretinal and sub-ILM spaces. Capillary macroaneurysms, however, have rarely presented with subretinal fluid. There may be several reasons for this difference. The first is simply difference in size. As mentioned before, when the vessel wall is weakened, the amount of dilatation is directly related to the parietal force on the vessel walls [9]. RAMs develop from the first three orders of high flow arterial circulation leading to larger aneurysms extending into the retina, whereas relatively, capillaries have much lower flow rates and thus develop dilatations of smaller caliber. Another consideration is intravascular hydrostatic pressure which must overcome potential retinal space; which is greater from fast flow arteries in comparison to slower flowing capillaries.

Our patient, however, presented with a foveal capillary macroaneurysm extending to the outer nuclear layer (ONL) with associated subretinal fluid. Blood-brain barrier disruption in exudative macroaneurysms results in a slowly prolonged leakage of plasma components through the weakened walls into the retina. Usually, this fluid spreads to the outer plexiform layer (OPL) and ONL where it concentrates due to the external limiting membrane (ELM) acting as a relative barrier to protein and osmotic forces [12]. The channels within the ELM, which is made up of zonular adherens between Müller cells and photoreceptors at the base of outer segments, are narrow and generally prevent fluid passage [12]. Any discontinuity of the ELM can result in flow of fluid from the intraretinal to subretinal space. In our patient, we hypothesize that there was a breach in the ELM at some point between his initial and follow-up visit presumably from intraretinal hydrostatic pressure of the intraretinal fluid observed on initial visit. Although follow-up OCT did not show any obvious breaks, they may be contained within the sections that were imaged. Another explanation is by analyzing macular anatomy [13]. Tsujikawa et al. observed a discontinuity in the outer retina when studying foveal architecture and also suggested that foveal Müller cells differ structurally from the rest of the retina. These differences, the authors predict, may allow for an easier path of fluid from the intraretina to the subretina [14]. Thus, we hypothesize that the juxtafoveal location of the macroaneurysm may play a part in the formation of subretinal fluid.

An important differential diagnosis is Perifoveal Exudative Vascular Anomalous Complex (PEVAC) defined by the presence of a unilateral, isolated perifoveal aneurysm in otherwise healthy individuals [15]. Our patient had stable mild nonproliferative diabetic retinopathy with scattered microaneurysms and hypertension, suggesting some degree of systemic vascular compromise, effectively ruling out PEVAC.

Capillary Macroaneurysms should be added to a differential diagnosis in cases of fusiform dilatation of vasculature in association with subretinal fluid. In our case, we hypothesize that the juxtafoveal location and discontinuity of the ELM played a part in the formation of subretinal fluid.

## Data Availability

Data sharing is not applicable to this article as no datasets were generated or analyzed during the current study.

## Abbreviations

OCT: Optical Coherence Tomography
FA: Fluorescein Angiography
ELM: External Limiting Membrane
RAM: Retinal Arterial Macroaneurysms
MMP9: Matrix Metallopeptidase 9

## References

[1] Kothari N, Mohsenin A, Medina C, Townsend J, Singh A (2016). Retinal Arterial Macroaneurysm. Manual of retinal diseases.

[2] Chew EY, Murphy RP, Ryan SJ, Schachat AP (2018). Aquired Retinal Macroaneurysms. Ryan’s Retina.

[3] Spaide RF, Barquet LA (2019). Retinal capillary macroaneurysms. Retina.

[4] Cousins SW, Flynn HW, Clarkson JG (1990). Macroaneurysms associated with retinal branch vein occlusion. Am J Ophthalmol.

[5] Farías DC (2019). Indocyanine green angiography for identifying Telangiectatic capillaries in diabetic macular edema. Br J Ophthalmol.

[6] Paques M, Philippakis E, Bonnet C, Falah S, Ayello-Scheer S, Zwillinger S (2016). Indocyanine-green-guided targeted laser photocoagulation of capillary macroaneurysms in macular oedema: a pilot study. Br J Ophthalmol.

[7] Parodi MB, Bondel E, Ravalico G (1995). Capillary Macroaneurysms in Central Retinal Vein Occlusion. *Italian Ministry of Health*, S. Karger AG, 18 Mar. 2016. Ophthalmologica.

[8] Stitt AW, Gardiner TA, Archer DB (1995). Histological and ultrastructural investigation of retinal microaneurysm development in diabetic patients. Br J Ophthalmol.

[9] Dubow M, Pinhas A, Shah N (2014). Classification of human retinal microaneurysms using adaptive optics scanning light ophthalmoscope fluorescein angiography. Invest Ophthalmol Vis Sci.

[10] Lόpez-Luppo M, Necher V, Ramos D (2017). Blood vessel basement membrane alterations in human retinal microaneurysms during aging. Invest Ophthalmol Vis Sci.

[11] Bourhis A, Girmens J-F, Boni S, Pecha F, Favard C, Sahel J-A, Paques M (2009). Imaging of macroaneurysms occurring during retinal vein occlusion and diabetic retinopathy by indocyanine green angiography and high resolution optical coherence tomography. Graefes Arch Clin Exp Ophthalmol.

[12] Marmor MF (2000). Mechanisms of fluid accumulation in retinal edema. Macular Edema.

[13] Ueda T, Gomi F, Suzuki M, Sakaguchi H, Sawa M, Kamei M, Nishida K (2012). Usefulness of Indocyanine green angiography to depict the distant retinal vascular anomalies associated with branch retinal vein occlusion causing serous macular detachment. Retina.

[14] Tsujikawa A (2010). Serous Retinal Detachment Associated With Retinal Vein Occlusion. Am J Ophthalmol.

[15] Sacconi R (2017). The Expanded Spectrum of Perifoveal Exudative Vascular Anomalous Complex. Am J Ophthalmol.

